# Prealbumin Prognostic Score: A Novel Prognostic Indicator After Radical Gastrectomy in Patients with Gastric Cancer

**DOI:** 10.3390/cancers16223889

**Published:** 2024-11-20

**Authors:** Ryota Matsui, Souya Nunobe, Motonari Ri, Rie Makuuchi, Tomoyuki Irino, Masaru Hayami, Manabu Ohashi, Takeshi Sano

**Affiliations:** Department of Gastroenterological Surgery, The Cancer Institute Hospital of Japanese Foundation for Cancer Research, Tokyo 135-8550, Japan; ryota.matsui@jfcr.or.jp (R.M.); motonari.ri@jfcr.or.jp (M.R.); rie.makuuchi@jfcr.or.jp (R.M.); tomoyuki.irino@jfcr.or.jp (T.I.); masaru.hayami@jfcr.or.jp (M.H.); manabu.ohashi@jfcr.or.jp (M.O.); takeshi.sano@jfcr.or.jp (T.S.)

**Keywords:** C-reactive protein, gastrectomy, prealbumin, prognosis, stomach neoplasms

## Abstract

**Background**: This study aimed to determine whether the prealbumin prognostic score (PPS), a novel indicator using prealbumin instead of albumin in the modified Glasgow Prognostic Score (mGPS), is a better predictive marker postoperatively in patients with gastric cancer. **Methods**: This retrospective study included consecutive patients who underwent radical gastrectomy for primary pStages I–III gastric cancer between 2006 and 2017. The cutoff values for preoperative prealbumin and C-reactive protein (CRP) were 22 mg/dL and 0.5 mg/dL, respectively. According to the prealbumin and CRP levels, a PPS of zero was defined as both being above the cutoff value, of one as either being below the cutoff value, and of two as both being below the cutoff value. **Results**: Of the 4663 patients, 3421 (73.4%) had a score of zero, 984 (21.1%) had a score of one, and 258 (5.5%) had a score of two. The higher the PPS, the poorer the overall survival [OS] (*p* < 0.001). When comparing OS by the PPS in patients with an mGPS of zero, a PPS of one indicated poorer OS than a PPS of zero (*p* < 0.001). In the multivariate analysis, PPSs of one (hazard ratio [HR]: 1.603; 95% confidence interval [CI]: 1.378–1.866; *p* < 0.001) and two (HR: 1.322; 95% CI: 1.055–1.656; *p* = 0.015) were independent poor prognostic factors for OS. **Conclusions**: The PPS, which is based on a combination of prealbumin and CRP levels, can identify a wider range of patients with poor OS than mGPS in patients with gastric cancer after gastrectomy.

## Simple Summary

The Glasgow Prognostic Score and modified Glasgow Prognostic Score using a combination of serum albumin and C-reactive protein have been reported in relation to postoperative prognosis in many cancers. In recent years, prealbumin has often been used as an alternative to albumin, but the prognostic impact of prealbumin-based indices in the above-mentioned indices has not yet been reported. We therefore developed the prealbumin prognostic score.

## 1. Introduction

The combination of serum albumin and C-reactive protein (CRP) is a useful risk factor for postoperative complications and a prognostic factor postoperatively for long-term survival in patients with gastric cancer [1,2,3,4]. The Glasgow Prognostic Score (GPS) and mGPS indices are based on CRP values [5,6]. These indices have been shown to be predictors of postoperative complications and poor prognostic factors for long-term survival in patients with gastric cancer [1,2,3,4]. When considering patients with gastric cancer for surgery, it is important for surgeons to identify those at high risk for poor short- and long-term postoperative outcomes. In particular, the identification of patients with a poor prognosis allows for enhanced perioperative management and the consideration of future enhanced therapy.

We found that prealbumin could more clearly distinguish patients with poorer long-term survival in patients with gastric cancer after radical gastrectomy than albumin [7]. Prealbumin is a marker with a shorter half-life than albumin and is used routinely in many institutions as a nutritional indicator [7,8]. Among patients with normal albumin levels, when divided into three groups according to prealbumin levels, the lower the prealbumin levels, the poorer the long-term survival [7]. Therefore, patients with normal albumin levels may have low prealbumin levels, leading to poor long-term survival. In contrast, prealbumin and albumin levels are inversely correlated with CRP levels [8]. Prealbumin alone can be used to identify patients with poor long-term survival; however, combining prealbumin with CRP may identify patients with a poorer prognosis.

This study aimed to determine whether the prealbumin prognostic score (PPS), a novel indicator using prealbumin instead of albumin, as used in mGPS, is a better predictive marker than mGPS. We hypothesized that patients with gastric cancer and a poor PPS would have poor long-term survival after gastrectomy.

## 2. Materials and Methods

### 2.1. Eligibility Criteria

This retrospective cohort study was conducted at the Cancer Institute Hospital of the Japanese Foundation for Cancer Research. This study included patients with primary gastric cancer who underwent radical gastrectomy between March 2006 and March 2017. The study included patients diagnosed with pStage I–III gastric cancer, according to the 15th edition of the Japanese Classification of Gastric Carcinoma [9]. Patients with remnant gastric cancer, multiple cancers, non-curative resection, or neoadjuvant chemotherapy were excluded. Clinical data, blood test results, and pathological findings were retrospectively collected from electronic medical records.

All experimental protocols outlined in this study were approved by the Institutional Ethical Review Committee (authorization number: 2023-GB-092). These protocols adhered to the ethical guidelines of the Japan Ministry of Health, Labour and Welfare for Medical and Health Research Involving Human Subjects and complied with the principles of the Declaration of Helsinki. Patient consent was obtained using an opt-out recruitment method.

### 2.2. Definitions

In accordance with previous reports, the cutoff values for preoperative prealbumin levels were set at 15 or 22 mg/dL [7]. Prealbumin levels were determined within 1 week prior to gastrectomy. The cutoff value for the preoperative CRP level was set to 0.5 mg/dL. According to prealbumin and CRP values, a PPS score of 0 was defined as both being above the cutoff value, a PPS score of 1 as either being below the cutoff value, and a PPS score of 2 as both being below the cutoff value. Similarly, with a cutoff value of 3.5 g/dL for albumin, an mGPS score of 0 was defined as both albumin and CRP being above the cutoff value, an mGPS score of 1 as either being below the cutoff value, and an mGPS score of 2 as both being below the cutoff value [6]. The definitions of each parameter are presented in Table 1. Patients were divided into subgroups based on preoperative prealbumin levels: high, >22 mg/dL; moderate, 15–22 mg/dL; and low, <15 mg/dL [7].

Postoperative complications were defined as those with a Clavien–Dindo classification [10] grade ≥ II occurring within 30 days postoperatively. Severe complications were defined as those with a Clavien–Dindo classification grade ≥ III.

### 2.3. Study Endopoints

The primary outcome measure was overall survival (OS), defined as the time between surgery and death. We compared OS in the three groups according to PPSs at prealbumin cutoffs of 15 and 22 mg/dL. For patients who were not followed up at our hospital, the hospital staff surveyed the public office five years postoperatively to determine whether the patient was alive or had died.

### 2.4. Perioperative Treatment

Patients who received neoadjuvant chemotherapy were excluded. Patients with pStage II–III gastric cancer received adjuvant chemotherapy with either tegafur/gimeracil/oteracil (S-1) or capecitabine plus oxaliplatin (XELOX), with dosage adjustments made as per the guidelines, if side effects occurred. S-1 and XELOX were administered for up to one year and six months, respectively. No additional treatments were administered until recurrence. Patients who experienced a relapse were treated according to the Japanese Gastric Cancer Treatment Guidelines [11,12].

Laparoscopic surgery was performed for patients with gastric cancer up to cT2, whereas open surgery was indicated for those with cancer stages higher than cT3. The lymph node dissection and reconstruction procedures were identical across both groups. D2 lymph node dissections or higher, as defined by the Japanese Gastric Cancer Treatment Guidelines, were classified as D2 [12]. Patients who underwent para-aortic lymph node dissection were excluded.

### 2.5. Statistical Analysis

The log-rank test was used for the Kaplan–Meier survival analysis of OS. First, OS was compared among the three groups according to the PPS. To determine the prognostic impact of prealbumin levels, OS was compared among the three groups according to prealbumin levels, dividing patients into those with low and high CRP levels. Finally, OS was compared among the three groups according to the mGPS, and OS was compared according to the PPS, focusing on patients with an mGPS score of 0. A Cox proportional hazards regression was used to identify prognostic factors, and multivariate analysis was conducted to calculate hazard ratios (HRs). Pearson’s correlation coefficient was used to examine the correlation between CRP and prealbumin levels. Receiver operating characteristic (ROC) curves were compared between PPS and mGPS to determine the best model that reflects OS. The Mann–Whitney U test was used to analyze continuous variables, while categorical variables were analyzed using Chi-squared or Fisher’s exact tests. All statistical analyses were performed using EZR software ver. 1.68 (Saitama Medical Center, Jichi Medical University, Saitama, Japan). A *p* value < 0.05 was deemed statistically significant.

## 3. Results

### 3.1. Characteristics of the Patients

Patient backgrounds according to the PPS with a prealbumin cutoff value of 15 mg/dL are presented in Table 2. Of the 4663 patients, 4190 (89.9%) had a score of zero, 386 (8.3%) had a score of one, and 87 (1.8%) had a score of two. Patients with higher PPSs were significantly older (*p* < 0.001) and had more advanced cStages and pStages (*p* < 0.001, both). Patient backgrounds according to the PPS with a prealbumin cutoff value of 22 mg/dL are presented in Table 3. Of the 4663 patients, 3421 (73.4%) were classified as having a score of zero, 984 (21.1%) as having a score of one, and 258 (5.5%) as having a score of two. Similarly, patients with higher PPSs were significantly older (*p* < 0.001) and had more advanced cStages and pStages (*p* < 0.001, both).

### 3.2. Comparison of OS

**Figure 1:**
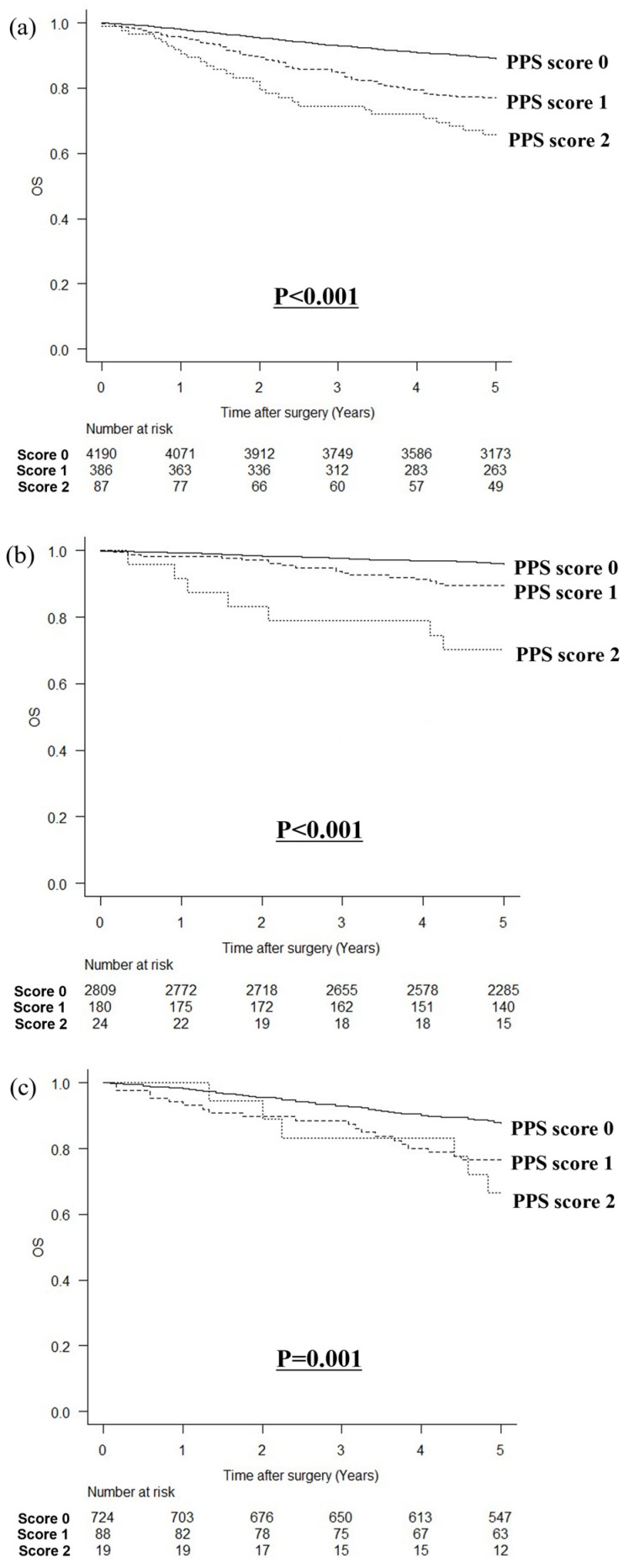

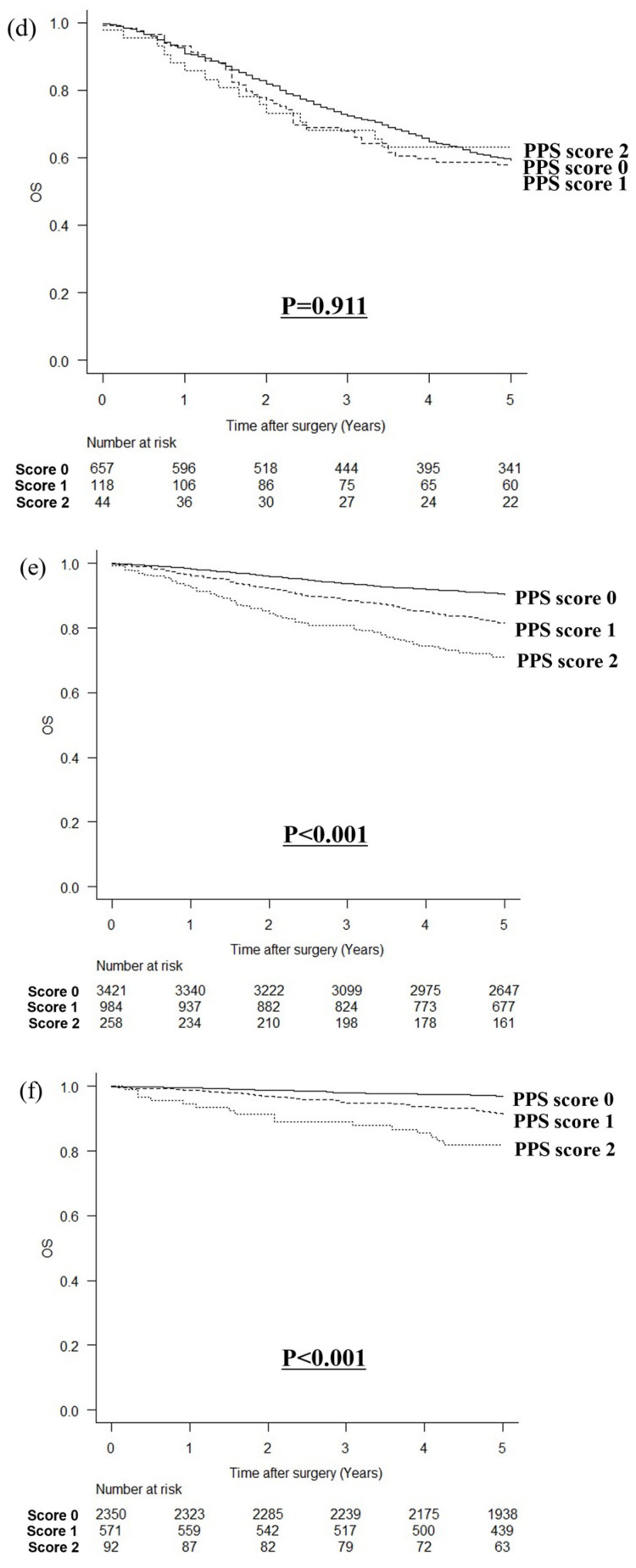

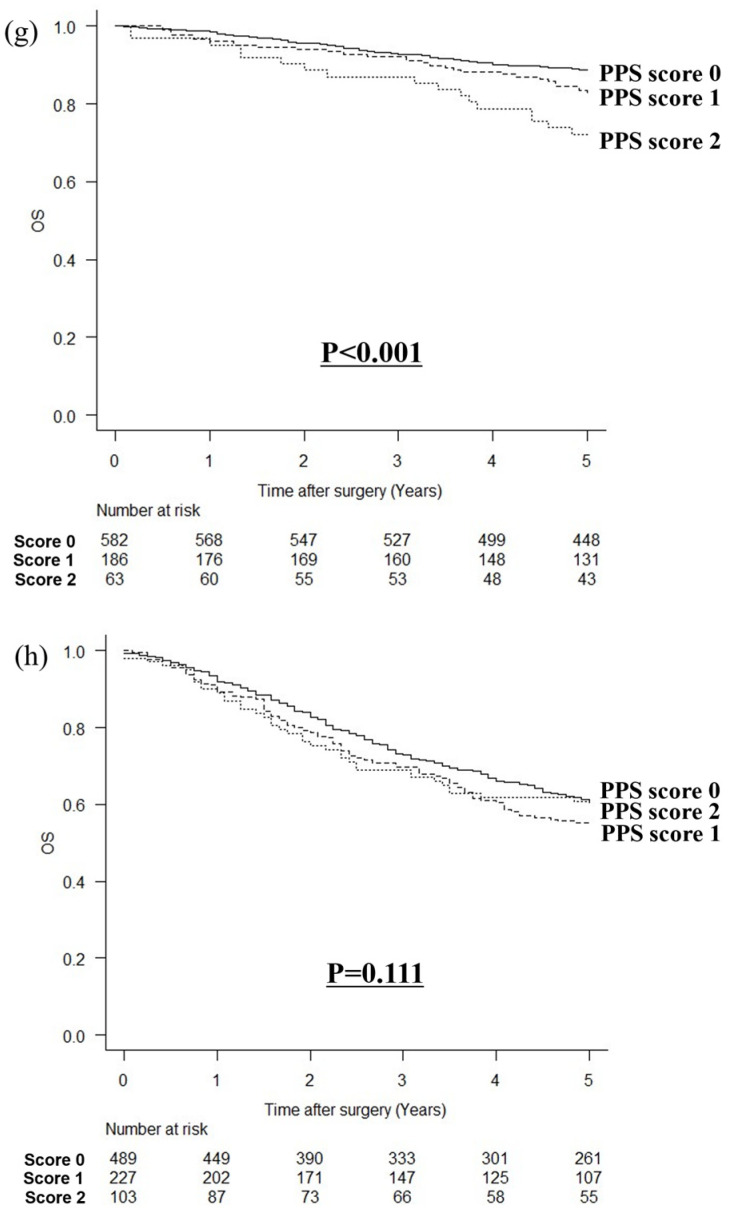
Relationship between PPS and overall survival. (**a**) PPS with a cutoff value of 15 mg/dL for prealbumin levels in all patients, (**b**) PPS with a cutoff value of 15 mg/dL for prealbumin levels in patients with pStage I cancer, (**c**) PPS with a cutoff value of 15 mg/dL for prealbumin levels in patients with pStage II cancer, (**d**) PPS with a cutoff value of 15 mg/dL for prealbumin levels in patients with pStage III cancer, (**e**) PPS with a cutoff value of 22 mg/dL for prealbumin levels in all patients, (**f**) PPS with a cutoff value of 22 mg/dL for prealbumin levels in patients with pStage I cancer, (**g**) PPS with a cutoff value of 22 mg/dL for prealbumin levels in patients with pStage II cancer, and (**h**) PPS with a cutoff value of 22 mg/dL for prealbumin levels in patients with pStage III cancer.

The median follow-up time, as indicated by the inclusion criteria, was 66 (interquartile range, 59–81) months. The relationship between the PPS and OS is shown in Figure 1. In a comparison of OS by the PPS with a cutoff value of 15 mg/dL for prealbumin, the higher the PPS, the poorer the OS (*p* < 0.001, Figure 1a). When stratified by the pStage, the higher the PPS, the worse the OS in stage I (*p* < 0.001, Figure 1b) or pStage II (*p* = 0.001, Figure 1c); however, there was no statistically significant difference in pStage III (*p* = 0.911, Figure 1d). In a comparison of OS by the PPS with a cutoff value of 22 mg/dL for prealbumin, the higher the PPS, the poorer the OS (*p* < 0.001, Figure 1e). Similarly, the higher the PPS, the worse the OS in stage I (*p* < 0.001, Figure 1f) and pStage II (*p* < 0.001, Figure 1g); however, there was no statistically significant difference in pStage III (*p* = 0.111, Figure 1h).

**Figure 2:**
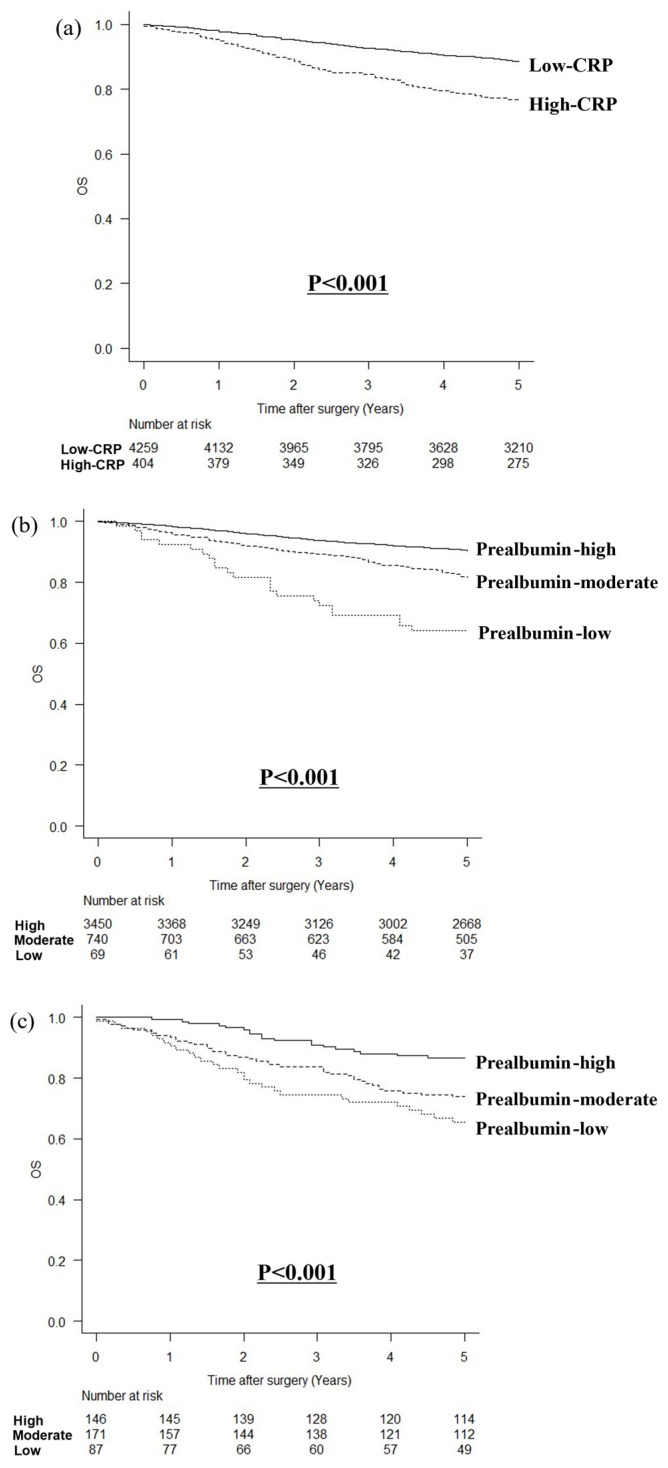
Comparison of overall survival according to CRP values and prealbumin levels. (**a**) Comparison of low-CRP and high-CRP groups, (**b**) according to prealbumin levels in patients with low CRP, and (**c**) according to prealbumin levels in patients with high CRP.

The CRP and prealbumin levels were inversely correlated (r = −0.292, *p* < 0.001). In a comparison of OS according to the CRP level, OS was poorer in patients with high CRP levels than in those with low CRP levels (*p* < 0.001; Figure 2a). In patients with low CRP levels, OS was poorer in those with lower prealbumin levels (*p* < 0.001; Figure 2b). Similarly, patients with high CRP levels had poorer OS and lower prealbumin levels (*p* < 0.001, Figure 2c).

**Figure 3:**
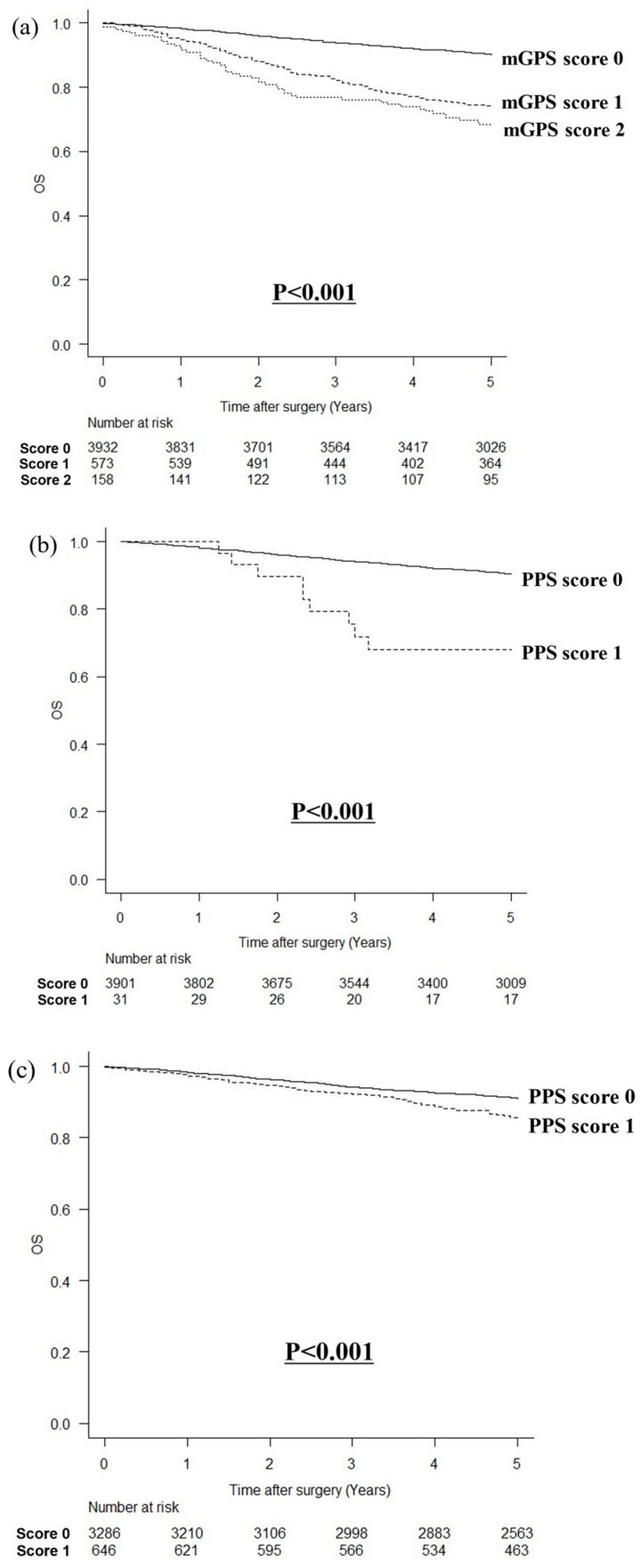
Comparison of overall survival according to mGPS and PPS. (**a**) According to the mGPS, (**b**) according to the PPS with a cutoff value of 15 mg/dL for prealbumin levels in patients with an mGPS of 0, and (**c**) according to the PPS with a cutoff value of 22 mg/dL for prealbumin levels in patients with an mGPS of 0.

In a comparison of OS by the mGPS, the higher the mGPS, the poorer the OS (*p* < 0.001, Figure 3a). When comparing OS by the PPS with a cutoff value of 15 mg/dL for prealbumin in patients with an mGPS score of zero, patients with a PPS score of one had poorer OS than those with a PPS score of zero (*p* < 0.001; Figure 3b). Similarly, comparing OS by the PPS with a cutoff value of 22 mg/dL for prealbumin in patients with an mGPS score of zero, patients with a PPS score of one had poorer OS than those with a PPS score of zero (*p* < 0.001; Figure 3c).

### 3.3. Comparison of RFS

**Figure 4:**
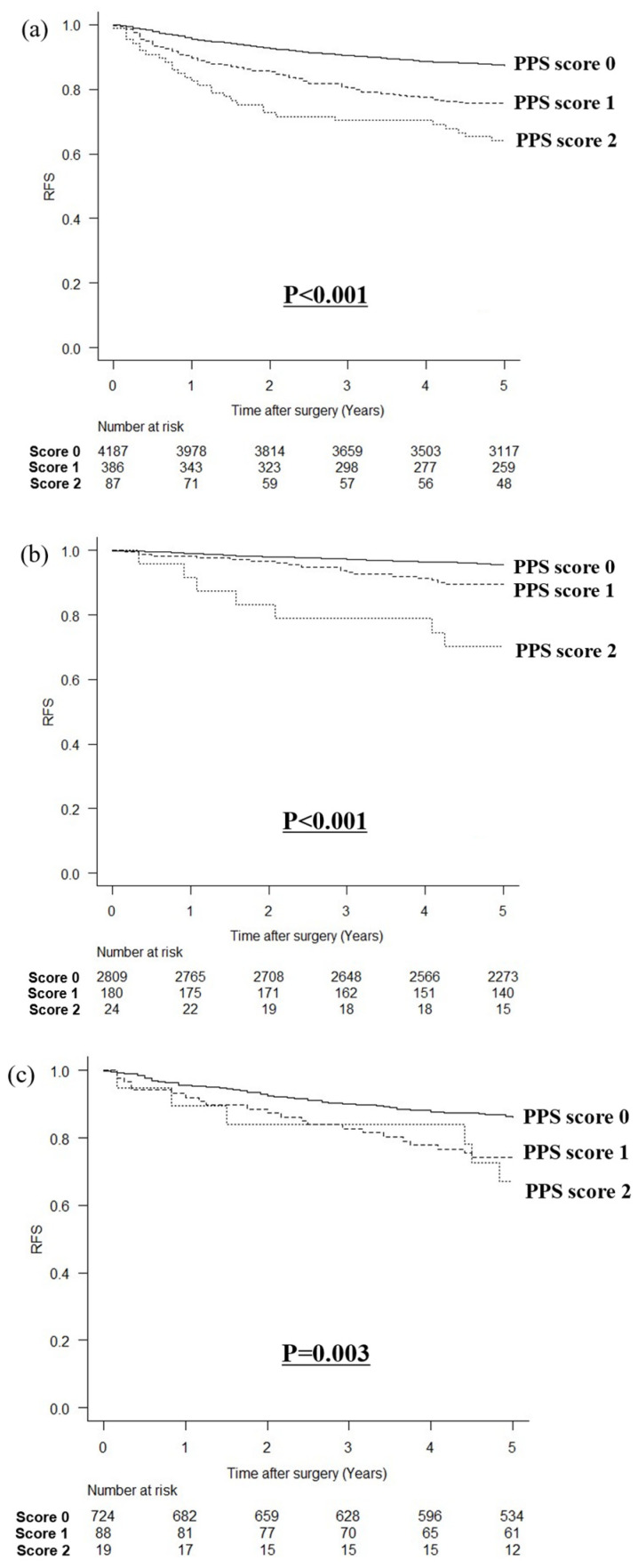

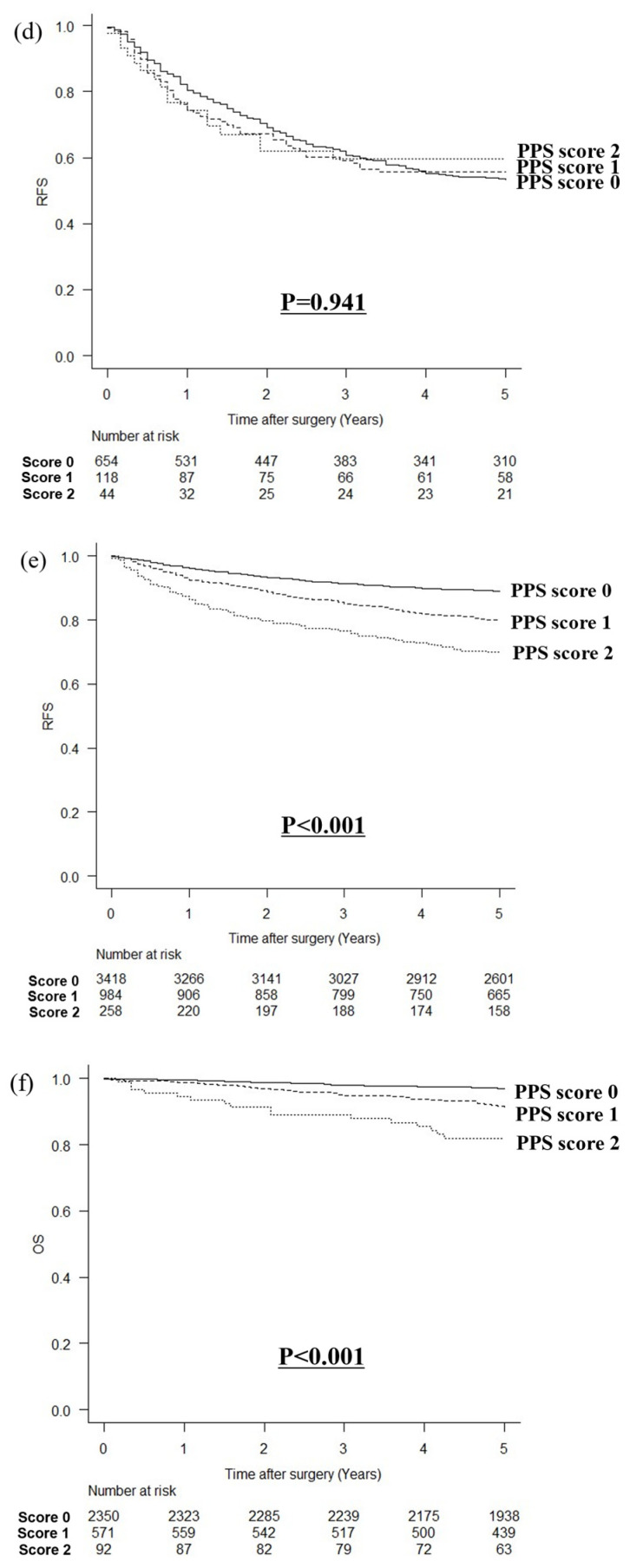

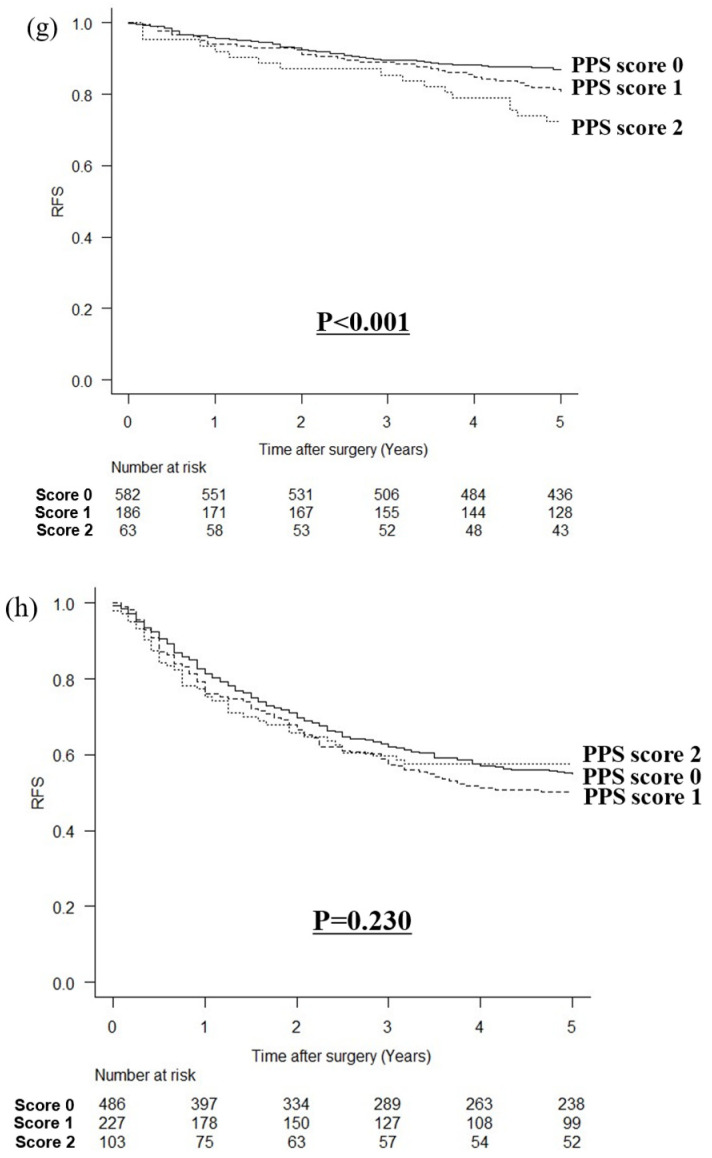
Relationship between PPS and recurrence-free survival. (**a**) PPS with a cutoff value of 15 mg/dL for prealbumin levels in all patients, (**b**) PPS with a cutoff value of 15 mg/dL for prealbumin levels in patients with pStage I cancer, (**c**) PPS with a cutoff value of 15 mg/dL for prealbumin levels in patients with pStage II cancer, (**d**) PPS with a cutoff value of 15 mg/dL for prealbumin levels in patients with pStage III cancer, (**e**) PPS with a cutoff value of 22 mg/dL for prealbumin levels in all patients, (**f**) PPS with a cutoff value of 22 mg/dL for prealbumin levels in patients with pStage I cancer, (**g**) PPS with a cutoff value of 22 mg/dL for prealbumin levels in patients with pStage II cancer, and (**h**) PPS with a cutoff value of 22 mg/dL for prealbumin levels in patients with pStage III cancer.

The relationship between the PPS and RFS is shown in Figure 4. In a comparison of RFS by the PPS with a cutoff value of 15 mg/dL for prealbumin, the higher the PPS, the poorer the RFS (*p* < 0.001, Figure 4a). When stratified by the pStage, the higher the PPS, the worse the RFS in stage I (*p* < 0.001, Figure 4b) or pStage II (*p* = 0.003, Figure 4c); however, there was no statistically significant difference in pStage III (*p* = 0.941, Figure 4d). In a comparison of RFS by the PPS with a cutoff value of 22 mg/dL for prealbumin, the higher the PPS, the poorer the RFS (*p* < 0.001, Figure 4e). Similarly, the higher the PPS, the worse the OS in stage I (*p* < 0.001, Figure 4f) and pStage II (*p* < 0.001, Figure 4g); however, there was no statistically significant difference in pStage III (*p* = 0.230, Figure 4h).

### 3.4. Prognostic Factors by Multivariate Analysis

The results of the multivariate analysis of the prognostic factors for OS according to the preoperative prealbumin cutoff values are shown in Table 4. In the multivariate analysis with a cutoff value of 15 mg/dL for prealbumin, a PPS score of two was an independent poor prognostic factor for OS (HR: 1.396; 95% confidence interval (CI): 1.010–1.928; *p* = 0.043). In the multivariate analysis with a cutoff value of 22 mg/dL for prealbumin, PPSs score of one (HR: 1.603; 95% CI: 1.378–1.866; *p* < 0.001) and two (HR: 1.322; 95% CI: 1.055–1.656; *p* = 0.015) were independent poor prognostic factors for OS.

The results of the multivariate analysis of the prognostic factors for OS according to preoperative CRP values are shown in Table 5. In the multivariate analysis of patients with CRP < 0.5 mg/dL, moderate (HR: 1.672; 95% CI: 1.414–1.976; *p* < 0.001) and low (HR, 1.806; 95% CI, 1.266–2.576; *p* = 0.001) prealbumin levels were independent poor prognostic factors for OS. In a multivariate analysis of patients with CRP ≥ 0.5 mg/dL, a low prealbumin level was an independent poor prognostic factor for OS (HR: 2.589; 95% CI: 1.520–4.409; *p* < 0.001).

The results of the multivariate analysis of the prognostic factors for OS according to the pStage are shown in Table 6. In the multivariate analysis of patients with pStage I and II cancer, a PPS score of one (HR: 2.086; 95% CI: 1.737–2.506; *p* < 0.001) and a PPS score of two (HR: 3.000; 95% CI: 2.269–3.967; *p* < 0.001) were independent poor prognostic factors for OS. However, in the multivariate analysis of patients with pStage III cancer, PPSs were not independent poor prognostic factors for OS.

The results of the multivariate analysis of the prognostic factors for RFS according to the preoperative prealbumin cutoff values are shown in Table 7. In the multivariate analysis with a cutoff value of 15 mg/dL for prealbumin, a PPS score of one or two was not an independent poor prognostic factor for RFS. In the multivariate analysis with a cutoff value of 22 mg/dL for prealbumin, PPSs score of one (HR: 1.520; 95% CI: 1.309–1.763; *p* < 0.001) and two (HR: 1.248; 95% CI: 1.000–1.558; *p* = 0.049) were independent poor prognostic factors for RFS.

### 3.5. Comparison of ROC Curves

**Figure 5:**
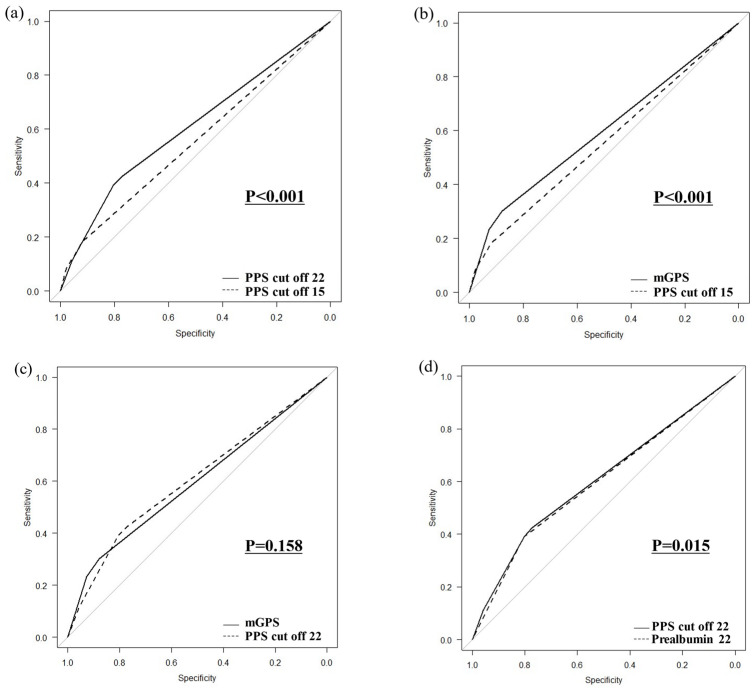
Comparison of ROC curves (**a**) between PPSs with prealbumin cutoff values of 15 mg/dL and 22 mg/dL, (**b**) between the PPS with a prealbumin cutoff of 15 mg/dL and the mGPS, (**c**) between the PPS with a prealbumin cutoff of 22 mg/dL and the mGPS, and (**d**) between the PPS with a prealbumin cutoff of 22 mg/dL and prealbumin alone.

In a comparison of ROC curves for PPSs with prealbumin cutoff values of 15 and 22 mg/dL, the PPS with a cutoff value of 22 mg/dL was more strongly correlated with OS (*p* < 0.001; Figure 5a). In a comparison of ROC curves between the PPS with a prealbumin cutoff of 15 mg/dL and the mGPS, the mGPS was more strongly correlated with OS (*p* < 0.001; Figure 5b). In a comparison of ROC curves of the PPS with a prealbumin cutoff of 22 mg/dL and the mGPS, no significant difference was found between the two indices (*p* = 0.158, Figure 5c). In a comparison of the PPS with a cutoff value of 22 mg/dL for prealbumin versus prealbumin alone, the PPS was more strongly associated with death (*p* = 0.015, Figure 5d).

## 4. Discussion

In this study, we determined the effect of the PPS, a new prognostic indicator that uses prealbumin instead of albumin in the mGPS, on OS after gastrectomy in patients with gastric cancer. OS was poorer with higher PPSs for both prealbumin cutoff values of 15 and 22 mg/dL. We observed several interesting findings in this study. First, in the multivariate analysis involving poor prognostic factors for OS, a PPS score of two was an independent poor prognostic factor at a prealbumin cutoff of 15 mg/dL, whereas both PPS scores of one and two were poor prognostic factors at a prealbumin cutoff of 22 mg/dL. Second, in a comparison of ROC curves, a prealbumin cutoff of 22 mg/dL was associated with a poorer OS than a cutoff of 15 mg/dL. Third, the ROC curve comparison showed no significant difference between the mGPS and PPSs with a cutoff value of 22 mg/dL, but the Kaplan–Meier curve showed that OS was worse in patients with a PPS score of one than in those with an mGPS score of zero. Fourth, when stratified by the pStage, the higher the PPS, the worse the OS in pStage I or pStage II; however, there was no statistically significant difference in pStage III. Fifth, OS was poorer with lower prealbumin levels in patients with both low and high CRP levels. Finally, in a comparison of ROC curves, the PPS with a cutoff value of 22 mg/dL for prealbumin was more strongly associated with poor OS than prealbumin alone. This study provides multifaceted evidence that the PPS, which reflects poor OS better than the mGPS and has been used to identify poor prognostic factors, is useful.

The cutoff value of CRP for the GPS has traditionally been 1.0 mg/dL [5,13], but a study in Japan proposed 0.5 mg/dL as a more optimal cutoff value for Asians and this has been used for the mGPS [6,14]. Higher CRP has been shown to be associated with a worse prognosis in patients with various cancers compared to those with normal CRP [15,16]. Lower albumin and prealbumin reflect both systemic inflammation and lean body mass [15,16]. Therefore, the GPS and PPS reflect patients with cachexia and may identify patients with poor long-term survival.

The cutoff value for the prealbumin level to be used in the PPS was 22 mg/dL. This is because, in a multivariate analysis related to poor prognostic factors for OS, a PPS score of two was an independent poor prognostic factor at a prealbumin cutoff of 15 mg/dL, whereas both PPSs of one and two were poor prognostic factors at a prealbumin cutoff of 22 mg/dL. Moreover, a comparison of ROC curves showed that a prealbumin cutoff of 22 mg/dL was associated with a poorer OS than a cutoff of 15 mg/dL. One reason for the better identification of patients with poor OS is that the corresponding patients with scores of one and two are different for each cutoff value. The percentage of patients with a PPS score of one or two was 10.1% for a prealbumin cutoff of 15 mg/dL compared to 26.6% for 22 mg/dL, which is a significant difference. Among patients with high or low CRP levels, OS was poorer with lower prealbumin levels; therefore, a cutoff value of 15 mg/dL for prealbumin may identify patients with the poorest prognosis, but a PPS score of one was not an independent prognostic factor in the multivariate analysis. Prognostic scores should be developed to more broadly identify patients with a poor prognosis so that more patients can be identified for the consideration of enhanced adjuvant chemotherapy and perioperative support. Accordingly, a prealbumin cutoff value of 22 mg/dL should be adopted as the value used in the PPS, which can broadly identify patients with a poor prognosis.

The PPS, with a cutoff value of 22 mg/dL, was shown to identify a wider range of patients with poorer OS than the mGPS. When comparing ROC curves, there was no significant difference between the mGPS and PPSs with a cutoff value of 22 mg/dL, but Kaplan–Meier curves showed that OS was worse in patients with a PPS score of one than in those with an mGPS score of zero. This may reflect the differential prognostic impact of albumin versus prealbumin. In a previous report, we have shown that when patients with albumin ≥ 3.5 g/dL were divided into three groups according to their prealbumin level, OS was poorer with lower prealbumin levels [7], indicating that even patients judged to have normal albumin levels by the mGPS have a poor prognosis if their prealbumin levels are <22 mg/dL. This may reflect the difference in half-life between albumin and prealbumin, with prealbumin being more sensitive to the effects of inflammation or undernutrition [7,8]. Therefore, the PPS proved to be able to identify a wider range of patients with a poor prognosis than the mGPS, which has been used for a long time.

The PPS combining prealbumin and CRP was more strongly associated with poor OS than prealbumin alone. In a comparison of ROC curves, the PPS with a cutoff value of 22 mg/dL for prealbumin was more strongly associated with poor OS than prealbumin alone. The proportion of patients with a prealbumin level < 22 mg/dL was 23.5%, whereas the proportion of patients with a high CRP level was 26.6%, indicating that a wider range of patients with a poorer prognosis could be selected. In this study, patients with high CRP levels had poorer OS than those with low CRP levels. The advantage of the PPS, which allows for the separate evaluation of each of the poor prognostic factors (CRP and prealbumin), is that patients with low levels of either can be more widely identified.

The PPS was not an independent prognostic factor for OS in patients with pStage III cancer. There are two possible reasons. First, the percentage of patients with a PPS score of one or two was approximately 20% in pStage I compared to 40% in pStage III. This indicates a higher prevalence of cachexia in patients with pStage III cancer. Second, patients with potential cachexia may be more common at pStage III. In this study, the more advanced the pStage, the higher the CRP and the lower the albumin and prealbumin. Although the PPS uses a cutoff value, it may be difficult to clearly separate patients with a poor prognosis when there are many patients with potential cachexia or cachexia as in pStage III.

As for the combination of CRP and prealbumin, these ratios have previously been shown to be associated with poor long-term survival postoperatively in patients with gastric or esophageal cancer [17,18,19], but no evidence-based cutoff values for each have been adopted. There is a wide range of cutoff values for the ratio of CRP to prealbumin, and the optimal cutoff value is optimal [17,18,19]. In contrast, the cutoff values for prealbumin of 22 mg/dL and CRP of 0.5 mg/dL have each been shown to be associated with a poor prognosis in previous reports [1,2,3,4,7]. The advantage of having two cutoff values for CRP and prealbumin is that it is easier to determine high-risk patients in daily clinical practice than using the ratio of the two.

This study had the limitation of being a single-center, retrospective study. To resolve this issue, further prospective multicenter studies examining the generalizability of these results are warranted. The strengths of this study are that it considered a large sample and applied cutoff values that are commonly used in daily practice. Therefore, these findings can be easily adapted in routine clinical practice. Patients with poor OS may require more intensive adjuvant chemotherapy and perioperative management, and the PPS should be used to identify these patients more extensively. The PPS may be used to identify early-stage patients with poorer OS, especially pStage I patients who require adjuvant chemotherapy. It may also be used as an indicator of the need for more intensive adjuvant chemotherapy than S-1 alone in patients with stage II disease. Further research is needed to prove the validity of this study in other clinical settings.

## 5. Conclusions

The PPS, a combination of prealbumin and CRP, can identify a wider range of patients with poor OS than the mGPS or prealbumin alone in patients with gastric cancer after gastrectomy. The cutoff value for prealbumin used in the PPS was 22 mg/dL to identify a wider range of patients with poor OS. This prognostic score should be developed to identify a wider range of patients with a poor prognosis and to identify patients who will require adjuvant chemotherapy, especially in pStage I, and those who will require more intensive adjuvant chemotherapy in pStage II.

## Figures and Tables

**Table 1:** Definitions of each parameter.

	mGPS	PPS: Cut Off 15mg/dL	PPS: Cut Off 15mg/dL
Score 0	Alb ≥ 3.5g/dL and CRP ≤ 0.5mg/dL	Prealbumin ≥ 15 mg/dL and CRP ≤ 0.5mg/dL	Prealbumin ≥ 22 mg/dL and CRP ≤ 0.5mg/dL
Score 1	Alb < 3.5g/dL or CRP > 0.5mg/dL	Prealbumin < 15 mg/dL or CRP > 0.5mg/dL	Prealbumin < 22 mg/dL or CRP > 0.5mg/dL
Score 2	Alb < 3.5g/dL and CRP > 0.5mg/dL	Prealbumin < 15 mg/dL and CRP > 0.5mg/dL	Prealbumin < 22 mg/dL and CRP > 0.5mg/dL

Alb albumin, CRP C-reactive protein, mGPS modified Glasgow Prognostic Score, PPS prealbumin prognostic score.

**Table 2:** Patient background according to PPS with a cutoff value of 15 mg/dL.

Prealbumin Prognostic Score (Prealbumin Cutoff Value: 15 mg/dL)	PPS Score 0 N = 4190	PPS Score 1 N = 386	PPS Score 2 N = 87	*p* Value
Age (years), Median (IQR)	64.0 (56.0, 72.0)	68.5 (60.3, 76.0)	71.0 (65.0, 79.0)	<0.001
Sex				0.378
Male	2687 (64.1%)	256 (66.3%)	51 (58.6%)	
Female	1503 (35.9%)	130 (33.7%)	36 (41.4%)	
Body mass index, Median (IQR)	22.5 (20.5, 24.6)	22.7 (20.2, 25.0)	21.0 (19.3, 22.8)	<0.001
Comorbidity				
Chronic kidney disease	548 (13.1%)	72 (18.7%)	18 (20.7%)	0.002
Diabetes	350 (8.4%)	53 (13.7%)	11 (12.6%)	0.001
CRP (mg/dL), Median (IQR)	0.08 (0.03, 0.10)	0.78 (0.51, 1.40)	2.10 (1.13, 4.75)	<0.001
Prealbumin (mg/dL), Median (IQR)	26.7 (23.1, 30.7)	20.3 (16.3, 24.4)	13.2 (10.9, 14.2)	<0.001
>22.0	3450 (82.3%)	146 (37.8%)	0 (0%)	
15.0–22.0	740 (17.7%)	171 (44.3%)	0 (0%)	<0.001
<15.0	0 (0%)	69 (17.9%)	87 (100%)	
Clinical stage				
I	3108 (74.2%)	194 (50.3%)	25 (28.7%)	<0.001
II	512 (12.2%)	69 (17.9%)	10 (11.5%)	
III	539 (12.9%)	117 (30.3%)	44 (50.6%)	
IVA	31 (0.7%)	6 (1.6%)	8 (9.2%)	
Surgical approach				
Laparoscopic surgery	2605 (62.2%)	145 (37.6%)	14 (16.1%)	<0.001
Open surgery	1585 (37.8%)	241 (62.4%)	73 (83.9%)	
Surgical procedure				
Distal gastrectomy	2396 (57.2%)	224 (58.0%)	55 (63.2%)	<0.001
Total gastrectomy	840 (20.0%)	118 (30.6%)	29 (33.3%)	
Proximal gastrectomy	195 (4.7%)	7 (1.8%)	1 (1.1%)	
Pylorus-preserving gastrectomy	759 (18.1%)	37 (9.6%)	2 (2.3%)	
Lymph node dissection				
D1+	2408 (57.5%)	145 (37.6%)	20 (23.0%)	<0.001
D2	1782 (42.5%)	241 (62.4%)	67 (77.0%)	
Serosal invasion	667 (15.9%)	153 (39.6%)	53 (60.9%)	<0.001
Lymph node metastasis				
N1	544 (13.0%)	64 (16.6%)	17 (19.5%)	<0.001
N2	369 (8.8%)	51 (13.2%)	19 (21.8%)	
N3	328 (7.8%)	62 (16.1%)	13 (14.9%)	
Pathological stage				
I	2809 (67.0%)	180 (46.6%)	24 (27.6%)	<0.001
II	724 (17.3%)	88 (22.8%)	19 (21.8%)	
III	657 (15.7%)	118 (30.6%)	44 (50.6%)	
Histological type				
Differentiated	1841 (43.9%)	179 (46.4%)	38 (43.7%)	0.651
Undifferentiated	2349 (56.1%)	207 (53.6%)	49 (56.3%)	
Postoperative complications				
Overall complications	820 (19.6%)	97 (25.1%)	20 (23.0%)	0.026
Severe complications	284 (6.8%)	30 (7.8%)	11 (12.6%)	0.085
Adjuvant chemotherapy	787 (18.8%)	110 (28.5%)	34 (39.1%)	<0.001

**Table 3:** Patient background according to the PPS with a cutoff value of 22 mg/dL.

Prealbumin Prognostic Score (Prealbumin Cutoff Value: 22 mg/dL)	PPS Score 0 N = 3421	PPS Score 1 N = 984	PPS Score 2 N = 258	*p* Value
Age (years), Median (IQR)	64.0 (56.0, 71.0)	68.0 (57.0, 76.0)	70.0 (64.0, 78.0)	<0.001
Sex				<0.001
Male	2377 (69.5%)	458 (46.5%)	159 (61.6%)	
Female	1044 (30.5%)	526 (53.5%)	99 (38.4%)	
Body mass index, Median (IQR)	22.7 (20.7, 24.7)	21.6 (19.4, 24.3)	21.5 (19.8, 24.1)	<0.001
Comorbidity				
Chronic kidney disease	449 (13.1%)	138 (14.0%)	51 (19.8%)	0.011
Diabetes	257 (7.5%)	120 (12.2%)	37 (14.3%)	<0.001
CRP (mg/dL), Median (IQR)	0.08 (0.03, 0.10)	0.10 (0.05, 0.30)	1.30 (0.74, 2.28)	<0.001
Prealbumin (mg/dL), Median (IQR)	28.1 (25.2, 31.5)	20.3 (18.1, 21.6)	17.1 (14.2, 19.8)	<0.001
>22.0	3421 (100.0%)	175 (17.8%)	0 (0%)	
15.0–22.0	0 (0%)	740 (75.2%)	171 (66.3%)	<0.001
<15.0	0 (0%)	69 (7.0%)	87 (33.7%)	
Clinical stage				
I	2599 (76.0%)	630 (64.0%)	98 (38.0%)	<0.001
II	406 (11.9%)	139 (14.1%)	46 (17.8%)	
III	397 (11.6%)	199 (20.2%)	104 (40.3%)	
IVA	19 (0.6%)	16 (1.6%)	10 (3.9%)	
Surgical approach				
Laparoscopic surgery	2198 (64.3%)	496 (50.4%)	70 (27.1%)	<0.001
Open surgery	1223 (35.7%)	488 (49.6%)	188 (72.9%)	
Surgical procedure				
Distal gastrectomy	1929 (56.4%)	593 (60.3%)	153 (59.3%)	<0.001
Total gastrectomy	679 (19.8%)	220 (22.4%)	88 (34.1%)	
Proximal gastrectomy	166 (4.9%)	33 (3.4%)	4 (1.6%)	
Pylorus-preserving gastrectomy	647 (18.9%)	138 (14.0%)	13 (5.0%)	
Lymph node dissection				
D1+	2009 (58.7%)	491 (49.9%)	73 (28.3%)	<0.001
D2	1412 (41.3%)	493 (50.1%)	185 (71.7%)	
Serosal invasion	477 (13.9%)	266 (27.0%)	130 (50.4%)	<0.001
Lymph node metastasis				
N1	433 (12.7%)	137 (13.9%)	55 (21.3%)	<0.001
N2	289 (8.4%)	108 (11.0%)	42 (16.3%)	
N3	236 (6.9%)	125 (12.7%)	42 (16.3%)	
Pathological stage				
I	2350 (68.7%)	571 (58.0%)	92 (35.7%)	<0.001
II	582 (17.0%)	186 (18.9%)	63 (24.4%)	
III	489 (14.3%)	227 (23.1%)	103 (39.9%)	
Histological type				
Differentiated	1507 (44.1%)	431 (43.8%)	120 (46.5%)	0.724
Undifferentiated	1914 (55.9%)	553 (56.2%)	138 (53.5%)	
Postoperative complications				
Overall complications	682 (19.9%)	194 (19.7%)	61 (23.6%)	0.339
Severe complications	233 (6.8%)	67 (6.8%)	25 (9.7%)	0.210
Adjuvant chemotherapy	630 (18.4%)	208 (21.1%)	93 (36.0%)	<0.001

**Table 4:** Results of analysis of prognostic factors for overall survival according to preoperative prealbumin cutoff values.

Variables	Prealbumin Cutoff Value: 15 mg/dL						Prealbumin Cutoff Value: 22 mg/dL					
	Univariate Analysis			Multivariate Analysis			Univariate Analysis			Multivariate Analysis		
	HR	95% CI	*p* Value	HR	95% CI	*p* Value	HR	95% CI	*p* Value	HR	95% CI	*p* Value
Sex												
Female	1			1			1			1		
Male	1.463	1.266–1.691	<0.001	1.482	1.279–1.719	<0.001	1.463	1.266–1.691	<0.001	1.582	1.362–1.838	<0.001
Age (years)												
<70	1			1			1			1		
≥70	2.988	2.619–3.408	<0.001	2.701	2.353–3.100	<0.001	2.988	2.619–3.408	<0.001	2.552	2.219–2.935	<0.001
Surgical procedure												
Non-TG	1			1			1			1		
TG	2.644	2.314–3.022	<0.001	1.597	1.383–1.846	<0.001	2.644	2.314–3.022	<0.001	1.591	1.378–1.836	<0.001
Surgical approach												
Laparoscopy	1			1			1			1		
Open	3.552	3.092–4.080	<0.001	2.323	1.844–2.926	<0.001	3.552	3.092–4.080	<0.001	2.164	1.719–2.723	<0.001
Lymph node dissection												
D1+	1			1			1			1		
D2	2.454	2.143–2.809	<0.001	0.693	0.553–0.868	0.001	2.454	2.143–2.809	<0.001	0.711	0.568–0.889	0.003
pStage												
I, II	1			1			1			1		
III	4.747	4.157–5.419	<0.001	3.300	2.737–3.977	<0.001	4.747	4.157–5.419	<0.001	3.228	2.676–3.893	<0.001
Histological type												
Differentiated	1			1			1			1		
Undifferentiated	0.856	0.751–0.975	0.019	0.988	0.861–1.134	0.866	0.856	0.751–0.975	0.019	0.990	0.863–1.136	0.884
Score												
0	1			1			1			1		
1	1.893	1.567–2.286	<0.001	1.113	0.916–1.353	0.283	1.905	1.656–2.192	<0.001	1.603	1.378–1.866	<0.001
2	3.087	2.256–4.224	<0.001	1.396	1.010–1.928	0.043	2.407	1.957–2.960	<0.001	1.322	1.055–1.656	0.015
Postoperative complication												
Absent	1			1			1			1		
Overall complications	1.603	1.385–1.856	<0.001				1.603	1.385–1.856	<0.001			
Severe complications	1.698	1.365–2.111	<0.001	1.268	1.016–1.583	0.036	1.698	1.365–2.111	<0.001	1.272	1.019–1.588	0.033
Adjuvant chemotherapy												
Absent	1			1			1			1		
Present	2.354	2.050–2.702	<0.001	0.894	0.747–1.069	0.218	2.354	2.050–2.702	<0.001	0.917	0.766–1.098	0.346

**Table 5:** Results of analysis of prognostic factors for overall survival according to preoperative CRP values.

Variables	CRP < 0.5 mg/dL					CRP ≥ 0.5 mg/dL						
	Univariate Analysis			Multivariate Analysis		Univariate Analysis				Multivariate Analysis		
	HR	95% CI	*p* Value	HR	95% CI	*p* Value	HR	95% CI	*p* Value	HR	95% CI	*p* Value
Sex												
Female	1			1			1			1		
Male	1.399	1.198–1.634	<0.001	1.541	1.310–1.812	<0.001	1.963	1.303–2.958	0.001	2.454	1.598–3.770	<0.001
Age (years)												
<70	1			1			1			1		
≥70	2.998	2.601–3.455	<0.001	2.705	2.325–3.148	<0.001	2.316	1.622–3.309	<0.001	1.794	1.235–2.606	0.002
Surgical procedure												
Non-TG	1			1			1			1		
TG	2.671	2.311–3.087	<0.001	1.653	1.415–1.930	<0.001	2.071	1.466–2.927	<0.001	1.616	1.087–2.404	0.018
Surgical approach												
Laparoscopy	1			1			1			1		
Open	3.570	3.079–4.139	<0.001	1.966	1.534–2.519	<0.001	2.443	1.608–3.712	<0.001	2.458	1.317–4.588	0.005
Lymph node dissection												
D1+	1			1			1			1		
D2	2.531	2.189–2.927	<0.001	0.757	0.594–0.964	0.024	1.443	0.994–2.095	0.054	0.536	0.308–0.936	0.028
pStage												
I, II	1			1			1			1		
III	5.176	4.484–5.975	<0.001	3.464	2.823–4.251	<0.001	2.284	1.614–3.232	<0.001	1.816	1.147–2.877	0.011
Histological type												
Differentiated	1			1			1			1		
Undifferentiated	0.862	0.749–0.992	0.038	0.986	0.849–1.146	0.857	0.831	0.590–1.172	0.291	0.949	0.661–1.362	0.777
Prealbumin (mg/dL)												
High	1			1			1			1		
Moderate	2.075	1.774–2.429	<0.001	1.672	1.414–1.976	<0.001	1.147	0.813–1.618	0.434	1.602	0.992–2.588	0.054
Low	3.756	2.676–5.273	<0.001	1.806	1.266–2.576	0.001	2.073	1.428–3.008	<0.001	2.589	1.520–4.409	<0.001
Postoperative complication												
Absent	1			1			1			1		
Overall complications	1.656	1.414–1.940	<0.001				1.169	0.791–1.729	0.434			
Severe complications	1.698	1.340–2.152	<0.001	1.242	0.976–1.581	0.078	1.645	0.941–2.875	0.081	1.424	0.798–2.540	0.231
Adjuvant chemotherapy												
Absent	1			1			1			1		
Present	2.512	2.163–2.916	<0.001	1.027	0.844–1.250	0.790	1.273	0.888–1.825	0.189	0.624	0.391–0.997	0.049

**Table 6:** Results of analysis of prognostic factors for overall survival according to pStage.

Variables	pStage I, II						pStage III					
	Univariate Analysis			Multivariate Analysis			Univariate Analysis			Multivariate Analysis		
	HR	95% CI	*p* Value	HR	95% CI	*p* Value	HR	95% CI	*p* Value	HR	95% CI	*p* Value
Sex												
Female	1			1			1			1		
Male	1.731	1.419–2.111	<0.001	1.929	1.567–2.376	<0.001	1.236	0.998–1.532	0.053	1.252	1.006–1.558	0.044
Age (years)												
<70	1			1			1			1		
≥70	4.859	4.059–5.817	<0.001	4.159	3.444–5.024	<0.001	1.432	1.165–1.759	<0.001	1.206	0.963–1.509	0.103
Surgical procedure												
Non-TG	1			1			1			1		
TG	2.311	1.923–2.777	<0.001	1.722	1.415–2.095	<0.001	1.439	1.176–1.760	<0.001	1.376	1.119–1.692	0.002
Surgical approach												
Laparoscopy	1			1			1			1		
Open	2.298	1.937–2.726	<0.001	1.679	1.303–2.164	<0.001	1.446	0.876–2.388	0.149	1.963	1.117–3.452	0.019
Lymph node dissection												
D1+	1			1			1			1		
D2	1.526	1.284–1.812	<0.001	0.797	0.621–1.023	0.075	0.590	0.404–0.863	0.006	0.480	0.314–0.734	<0.001
Histological type												
Differentiated	1			1			1			1		
Undifferentiated	0.594	0.499–0.707	<0.001	0.957	0.780–1.147	0.631	0.972	0.783–1.205	0.794	1.140	0.913–1.423	0.249
Score (Prealbumin cutoff: 15)												
0	1			1			1			1		
1	2.086	1.737–2.506	<0.001	2.027	1.664–2.469	<0.001	1.258	1.010–1.565	0.040	1.212	0.957–1.534	0.110
2	3.000	2.269–3.967	<0.001	2.535	1.879–3.420	<0.001	0.981	0.720–1.335	0.902	0.962	0.692–1.337	0.817
Postoperative complication												
Absent	1			1			1			1		
Overall complications	1.480	1.212–1.808	<0.001				1.250	1.006–1.553	0.044			
Severe complications	1.535	1.128–2.091	0.006	1.194	0.873–1.632	0.267	1.444	1.061–1.966	0.020	1.269	0.923–1.746	0.143
Adjuvant chemotherapy												
Absent	1			1			1			1		
Present	1.848	1.454–2.349	<0.001	1.706	1.313–2.217	<0.001	0.520	0.423–0.640	<0.001	0.564	0.453–0.701	<0.001

**Table 7:** Results of analysis of prognostic factors for recurrence-free survival according to preoperative prealbumin cutoff values.

Variables	Prealbumin Cutoff Value: 15 mg/dL						Prealbumin Cutoff Value: 22 mg/dL					
	Univariate Analysis			Multivariate Analysis			Univariate Analysis			Multivariate Analysis		
	HR	95% CI	*p* Value	HR	95% CI	*p* Value	HR	95% CI	*p* Value	HR	95% CI	*p* Value
Sex												
Female	1			1			1			1		
Male	1.415	1.229–1.629	<0.001	1.416	1.226–1.636	<0.001	1.415	1.229–1.629	<0.001	1.498	1.294–1.733	<0.001
Age (years)												
<70	1			1			1			1		
≥70	2.883	2.534–3.279	<0.001	2.607	2.277–2.985	<0.001	2.883	2.534–3.279	<0.001	2.479	2.161–2.843	<0.001
Surgical procedure												
Non-TG	1			1			1			1		
TG	2.662	2.336–3.035	<0.001	1.542	1.339–1.776	<0.001	2.662	2.336–3.035	<0.001	1.542	1.339–1.775	<0.001
Surgical approach												
Laparoscopy	1			1			1			1		
Open	3.719	3.245–4.263	<0.001	2.291	1.824–2.877	<0.001	3.719	3.245–4.263	<0.001	2.149	1.712–2.698	<0.001
Lymph node dissection												
D1+	1			1			1			1		
D2	2.602	2.277–2.972	<0.001	0.717	0.574–0.895	0.003	2.602	2.277–2.972	<0.001	0.733	0.588–0.915	0.006
pStage												
I, II	1			1			1			1		
III	5.191	4.560–5.910	<0.001	3.409	2.844–4.085	<0.001	5.191	4.560–5.910	<0.001	3.334	2.781–3.998	<0.001
Histological type												
Differentiated	1			1			1			1		
Undifferentiated	0.861	0.758–0.979	0.022	0.959	0.838–1.098	0.543	0.861	0.758–0.979	0.022	0.961	0.840–1.100	0.565
Score												
0	1			1			1			1		
1	1.877	1.560–2.259	<0.001	1.090	0.900–1.320	0.376	1.855	1.617–2.129	<0.001	1.520	1.309–1.763	<0.001
2	3.018	2.214–4.113	<0.001	1.297	0.943–1.784	0.110	2.368	1.931–2.903	<0.001	1.248	1.000–1.558	0.049
Postoperative complication												
Absent	1			1			1			1		
Overall complications	1.578	1.367–1.823	<0.001				1.578	1.367–1.823	<0.001			
Severe complications	1.761	1.426–2.176	<0.001	1.310	1.056–1.624	0.014	1.761	1.426–2.176	<0.001	1.318	1.063–1.634	0.012
Adjuvant chemotherapy												
Absent	1			1			1			1		
Present	2.585	2.260–2.956	<0.001	0.989	0.831–1.176	0.898	2.585	2.260–2.956	<0.001	1.011	0.850–1.204	0.898

## Data Availability

The datasets generated and/or analyzed during the current study are available upon reasonable request from the corresponding author.

## References

[1] Shimoda Y., Fujikawa H., Komori K., Watanabe H., Takahashi K., Kano K., Yamada T., Shiozawa M., Morinaga S., Katsumata K. (2022). The Glasgow Prognostic Score Before Curative Resection May Predict Postoperative Complications in Patients with Gastric Cancer. J. Gastrointest. Cancer.

[2] Jiang X., Hiki N., Nunobe S., Kumagai K., Kubota T., Aikou S., Sano T., Yamaguchi T. (2012). Prognostic importance of the inflammation-based Glasgow prognostic score in patients with gastric cancer. Br. J. Cancer.

[3] Wang D.-S., Ren C., Qiu M.-Z., Luo H.-Y., Wang Z.-Q., Zhang D.-S., Wang F.-H., Li Y.-H., Xu R.-H. (2012). Comparison of the prognostic value of various preoperative inflammation-based factors in patients with stage III gastric cancer. Tumour Biol..

[4] Dutta S., Crumley A.B., Fullarton G.M., Horgan P.G., McMillan D.C. (2012). Comparison of the prognostic value of tumour and patient related factors in patients undergoing potentially curative resection of gastric cancer. Am. J. Surg..

[5] Forrest L.M., McMillan D.C., McArdle C.S., Angerson W.J., Dunlop D.J. (2003). Evaluation of cumulative prognostic scores based on the systemic inflammatory response in patients with inoperable non-small-cell lung cancer. Br. J. Cancer.

[6] Toiyama Y., Miki C., Inoue Y., Tanaka K., Mohri Y., Kusunoki M. (2011). Evaluation of an inflammation-based prognostic score for the identification of patients requiring postoperative adjuvant chemotherapy for stage II colorectal cancer. Exp. Ther. Med..

[7] Matsui R., Ida S., Ri M., Makuuchi R., Hayami M., Kumagai K., Ohashi M., Sano T., Nunobe S. (2024). Impact of preoperative prealbumin levels on long-term prognosis in patients with gastric cancer after gastrectomy: A retrospective cohort study. Gastric Cancer.

[8] Evans D.C., Corkins M.R., Malone A., Miller S., Mogensen K.M., Guenter P., Jensen G.L., The ASPEN Malnutrition Committee (2021). The Use of Visceral Proteins as Nutrition Markers: An ASPEN Position Paper. Nutr. Clin. Pract..

[9] Nakamura T., Hojo Y., Kumamoto T., Kurahashi Y., Ishida Y., Shinohara H. (2022). History of the lymph node numbering system in the Japanese Classification of Gastric Carcinoma since 1962. Surg. Today.

[10] Katayama H., Kurokawa Y., Nakamura K., Ito H., Kanemitsu Y., Masuda N., Tsubosa Y., Satoh T., Yokomizo A., Fukuda H. (2016). Extended Clavien-Dindo classification of surgical complications: Japan Clinical Oncology Group postoperative complications criteria. Surg. Today.

[11] Japanese Gastric Cancer Association (2017). Japanese gastric cancer treatment guidelines 2014 (ver. 4). Gastric Cancer.

[12] Japanese Gastric Cancer Association (2021). Japanese gastric cancer treatment guidelines 2018 (5th edition). Gastric Cancer.

[13] Forrest L.M., McMillan D.C., McArdle C.S., Angerson W.J., Dunlop D.J. (2004). Comparison of an inflammation-based prognostic score (GPS) with performance status (ECOG) in patients receiving platinum-based chemotherapy for inoperable non-small-cell lung cancer. Br. J. Cancer.

[14] Inoue Y., Iwata T., Okugawa Y., Kawamoto A., Hiro J., Toiyama Y., Tanaka K., Uchida K., Mohri Y., Miki C. (2013). Prognostic significance of a systemic inflammatory response in patients undergoing multimodality therapy for advanced colorectal cancer. Oncology.

[15] McMillan D.C. (2009). Systemic inflammation, nutritional status and survival in patients with cancer. Curr. Opin. Clin. Nutr. Metab. Care.

[16] McMillan D.C. (2008). An inflammation-based prognostic score and its role in the nutrition-based management of patients with cancer. Proc. Nutr. Soc..

[17] Maruyama S., Okamura A., Kanie Y., Sakamoto K., Fujiwara D., Kanamori J., Imamura Y., Kumagai K., Watanabe M. (2022). C-reactive protein to prealbumin ratio: A useful inflammatory and nutritional index for predicting prognosis after curative resection in esophageal squamous cell carcinoma patients. Langenbeck’s Arch. Surg..

[18] Aoyama T., Hashimoto I., Maezawa Y., Hara K., Komori K., Otani K., Kazama K., Sawazaki S., Numata M., Kamiya N. (2023). The C-reactive Protein to Prealbumin Ratio is an Independent Prognostic Factor for Patients with Gastric Cancer Who Receive Curative Surgery. Anticancer Res..

[19] Lu J., Xu B.-B., Zheng Z.-F., Xie J.-W., Wang J.-B., Lin J.-X., Chen Q.-Y., Cao L.-L., Lin M., Tu R.-H. (2019). CRP/prealbumin, a novel inflammatory index for predicting recurrence after radical resection in gastric cancer patients: Post hoc analysis of a randomized phase III trial. Gastric Cancer.

